# Knowledge and Perceptions of Greek Students about Human Papilloma Virus, Vaccination and Cervical Cancer Screening

**DOI:** 10.3390/children9121807

**Published:** 2022-11-24

**Authors:** Panagiota Koutrakou, Maria Trigoni, Pavlos Sarafis, Chara Tzavara, Athanasios Nikolentzos, Tonia Vassilakou, Theodoros N. Sergentanis

**Affiliations:** 1 School of Social Sciences, Hellenic Open University, 26335 Patras, Greece; 2 General Department Lamia, University of Thessaly, 35100 Lamia, Greece; 3 Centre for Health Services Research, Department of Hygiene, Epidemiology and Medical Statistics, Medical School, National and Kapodistrian University of Athens, 11527 Athens, Greece; 4 Department of Public Health Policies, School of Public Health, University of West Attica, 11521 Athens, Greece

**Keywords:** health literacy, youth, adolescents, cervical cancer, vaccination

## Abstract

INTRODUCTION: Human papillomavirus (HPV) is the main cause of cervical cancer; the level of HPV-related knowledge among young students remains however questionable. The purpose of the present study was to investigate knowledge pertaining to HPV, cervical cancer screening, and vaccination among students in the Nursing Department and Department of Social Work of the Hellenic Mediterranean University of Crete, Greece. METHODS: This was a questionnaire-based, cross-sectional study involving 371 first-year and third-year students of the two Departments. Multivariate linear and logistic regression analysis was performed to examine factors associated with knowledge related to HPV, cervical cancer screening, and HPV vaccination. RESULTS: Only 22.1% of students knew all the ways of HPV transmission and only 5.9% knew the whole spectrum of cancers that HPV could cause. The vaccination rate for HPV was 33.7%. The majority of students used the Internet as the main source of information (62.3%). Students’ sociodemographic characteristics, including age, marital status, and Department of studies were associated with knowledge about HPV. CONCLUSIONS: The present study highlights knowledge gaps and indicates the need for thorough health education strategies on HPV, targeting families and young people.

## 1. Introduction

Human Papilloma Virus (HPV) is a small DNA virus responsible for the pathogenesis of cervical cancer, the fourth most common cancer in women worldwide [1]. HPV infection is associated with the development of cancers of the vagina, vulva, anus, penis, oral cavity, tonsils, tongue, pharynx, and larynx [2]. HPV is the commonest sexually transmitted infection among young people [3]. It is mainly transmitted through direct contact of the genitals during vaginal, oral, and anal contact, but also through the contact of the skin of the sufferer with healthy skin [4]. According to the World Health Organization, 75% of sexually active people (men and women) will be infected with the virus at least once, while others will be infected repeatedly [3]. The beginning of sex life at an early age, the frequent exchange of partners, unsafe sexual behaviors, social profile, and smoking, appear to be associated with an increased risk of infection with the virus [5,6,7,8].

Vaccination is the most effective and safe method of primary prevention of cervical cancer and HPV infection worldwide [9]. Secondary prevention is also a particularly important factor in reducing cervical cancer mortality, through participation in organized screening programs or, in countries where such programs are not available (as is the case of Greece) at the individual level [10]. Pap smears and HPV DNA tests, are primary screening tests [11]. In Greece, cervical cancer is the twelfth most common cancer in women and the fourth most common in women aged 15–44 [8].

Worldwide research data on students’ knowledge and attitudes about HPV and its consequences have indicated inadequate student information on the virus, low vaccine coverage, and unsafe sexual practices. The purpose of the present study was to investigate the knowledge of students in two Departments (Nursing; Social Work) of the Hellenic Mediterranean University of Crete about HPV, screening tests and vaccination. In light of the above, the present study aimed to investigate the hypothesis of whether sociodemographic variables (age, gender, year of study, Department of studies, marital status) are related to the knowledge and perception of Greek students about HPV, vaccination, and cervical cancer screening. The absence of research data and studies about students’ knowledge and attitudes about HPV which are related to sociodemographic characteristics in two different Departments (Nursing; Social Work), in Greece and especially in the region of Crete, makes the present study very useful for other cross-national comparative studies.

## 2. Materials and Methods

### 2.1. Overall Design and Ethical Issues

The present study was conducted among first-year and third-year students of the Nursing Department and Department of Social Work of the Hellenic Mediterranean University of Crete, from February to March 2019. For the purpose of conducting the study, the research protocol was drafted and the relevant authorization for the research was obtained from the local Institutional Review Boards.

The research was carried out on a voluntary basis after an oral briefing on the purpose of the research and the content of the questions. The criteria for participation in the study were to be first- or third-year students of the Department of Nursing or the Department of Social Work to investigate how age might affect the knowledge and perceptions of Greek students at the beginning and at the end of their studies, and how the Department of studies (Nursing or Social Work is associated with knowledge about HPV.

The distribution of the questionnaires took place in the classrooms before the start or at the end of the theoretical lectures and workshops. The time given for completion was 10–15 min. The questionnaire was strictly personal, anonymous, the confidentiality of the elements was respected and all ethical requirements were fulfilled, and as provided by legislation.

### 2.2. Study Sample

The study population was all first-year and third-year students of the Nursing Department and Department of Social Work, Hellenic Mediterranean University of Crete, Greece. The sample consisted of 371 students of both genders, namely 195 students from the Nursing Department and 176 students from the Social Work Department. The majority of students who participated in this study had a Greek nationality and some of them had other nationalities.

A total of 372 questionnaires were distributed, of which 372 were collected and only one (*n* = 1) was not completed. On the basis of power calculation, a sample size of 372 persons is sufficient to achieve 90% power when detecting a difference of 5 points in the knowledge scores for equally sized subgroups, assuming a standard deviation of 15 points, in a two-tailed test and statistical significance level set at 0.05. Power calculation was performed with G * Power 3.1.9.2 software (University of Düsseldorf, Düsseldorf, Germany).

### 2.3. Instrument

The tool was a structured, anonymous, validated questionnaire by Dafermou C. et al. [12]; for its use permission was obtained from its creator. The questionnaire included 67 questions out of which 4 have been removed. The first 9 questions related to sociodemographic characteristics (age, nationality, marital status, gender, education, educational level, number of brothers, and maternal/paternal education level).

From the questions included in the questionnaire, some were closed items, while others were open, related to clarifying precautionary measures by the respondents, symptoms of the virus they developed, and any medications received. The questionnaire included questions that were addressed separately to each gender while some of them were common to both genders. The questionnaire consisted of 13 knowledge questions about HPV per se, 33 questions about knowledge and attitudes towards the HPV vaccine, 11 questions about screening tests addressed only to females, 2 questions addressed only to males, and finally 5 questions related to sexual behaviors and attitudes.

Given the changes in both the vaccination guidelines and the approval in Greece of the 9-Valent HPV vaccine against, a multiple-choice option pertaining to the vaccine 9vHPV was added to the question about the vaccine type students had been vaccinated with. Finally, a question about the HPV-DNA test was added, given the recent progress in cervical cancer screening.

### 2.4. Statistical Analysis

The collected data were analyzed using SPSS 22.0 statistical software (IBM. Corp., Armonk, NY, USA). Knowledge scores were calculated, as appropriate. Mean, standard deviation (SD) and median, and interquartile ranges were used to describe the numeric variables, as appropriate. Absolute (n) and relative (%) frequencies were used to describe the categorical variables. Multivariate linear regression analysis was used to evaluate independent factors related to the knowledge scores about HPV per se, HPV vaccination, and cervical cancer screening (the latter analysis was confined to females); regression coefficients (b) and their standard errors (SE) were estimated. Linear regression models were implemented using logarithmic transformations of the dependent variable (score). Significance levels were two-sided and statistical significance was set at 0.05.

## 3. Results

### 3.1. Description of the Study Sample

The sample consisted of 371 students. A total of 83.6% of the participants were females and 16.4% were males. The majority of students (80.9%) were 18–21 years old. Almost all participants (92.4%) were Greek and only 7.6% had other nationalities. A total of 52.7% of students were studying in the Nursing Department and the remaining percentage (47.3%) in the Department of Social Work; 54.1% of students were first-year students, while 45.9% were third-year students. The majority of participants (65.4%) were single. The majority of students had parents with 7–12 years of education (Table 1).

### 3.2. Knowledge Scores

Regarding students’ knowledge of HPV, rates of correct answers varied from 3.0% to 83.8% with the average knowledge score being 43.5% (SD = 18.7%) (Table 2). The average score of knowledge about the vaccine reached 28.4% (SD = 16.2%), 6.2% of the students *(n* = 23) did not answer any of the questions correctly (they scored zero), while no one answered them all correctly. The average knowledge score on female screening reached 76.1% (SD = 19.3%). 1.9% of students (*n* = 6) did not answer any of the questions correctly (they scored zero) and 20.0% (*n* = 62) answered them all correctly (Table 2).

Specifically, only 5.9% knew the whole spectrum of cancers the HPV infection could cause. The percentage of students who were aware of the possible ways virus transmission (22.1%) and the ways of preventing HPV (19.4%) was particularly low. However, the majority of the participants (75.9%) knew what HPV is, having been informed by the Internet (62.3%) (Table 3).

### 3.3. Associations with Knowledge Score about HPV

Table 4 shows the results of multivariate linear regression with the dependent variable the score of knowledge for HPV. Student’s age, education, and marital status were associated with the HPV knowledge score. Specifically, students over the age of 21 had a significantly higher score, that is, better knowledge about HPV compared to students who were 18–21 years of age. Students in the Department of Social Work had significantly lower score compared to Nursing students and, finally, students who were not married scored significantly lower that married ones (Table 3).

### 3.4. Associations with Knowledge Score about HPV Vaccination

The vaccination rate for HPV was equal to 33.7% (Table 5). Prompting of their family (68.3%) was the main reason. However, 69.5% were aware of the vaccine’s existence (*n* = 258), having been primarily informed by the Internet (45.7%, 108/258). Among participants who had not been vaccinated, the majority did not proceed to vaccination because of insufficient information (*n* = 135), followed by concerns for side effects (*n* = 41), declared absence of sexual contacts (*n* = 37), reported negative attitude by the physicians (*n* = 16) and fear that the vaccine could transmit the virus (*n* = 5).

According to the results of multivariate linear regression, nationality and education were associated with HPV vaccination knowledge scores. Specifically, students of non-Greek nationalities had significantly lower scores, that is lower knowledge about the HPV vaccine compared to Greek students; accordingly, the students of the Department of Social Work scored lower than the students of the Department of Nursing (Table 6).

### 3.5. Associations with Knowledge Score about Cervical Cancer Screening

Among female students who participated in the study, 45.5% (*n* = 138) reported going once a year to their gynecologist and 39.3% had a Pap smear test. The main reason they did not have a Pap test was the absence of sex contacts (*n* = 92, 49.2%). More than two-thirds (*n* = 90, 73.8%) did the test once a year and 9.3% (*n* = 11) had one test positive for HPV infection. The majority (97.7%, *n* = 298) knew what the “Pap smear test” is, 79.7% (*n* = 244) knew it was aimed at the detection of precancerous lesions, while only 23.7% knew what HPV-DNA test is (*n* = 71). Regarding male students, 49.2% (*n* = 29) of them had visited a specialist doctor for genital examination.

Age and Department of studies were independently related to the knowledge score on screening tests according to the results of multivariate linear regression (Table 7). Specifically, female students over the age of 21 had a significantly higher score, that is, better knowledge of screening test, compared to students who were 18–21 years old, while students in the Department of Social Work scored significantly lower, compared to Nursing students.

The Department of studies was found to be significantly related to female students having a Pap smear test according to the results of the multivariate logistic regression analysis (Table 8).

### 3.6. Sexual Behaviors and Attitudes

Based on the results of the study, the majority of the students in both Departments (*n* = 278, 85.3%) were taking precautions during sexual intercourse, using a condom (*n* = 192, 96%). A high percentage of students (*n* = 272, 85.0%) stated that they would like to be vaccinated with any of the existing vaccines to protect against HPV.

## 4. Discussion

The results of this study showed significant associations regarding the knowledge and perceptions of students about HPV, screening tests and vaccination. In particular, 75.9% of participants stated that they knew what HPV is, declaring that they had been informed by the Internet. Similar rates have been observed by Lopez and McMahan (79.5%), Gerend and Magloire (75%), and finally in a Greek study by Nanou et al. (70.7%) [13,14,15]. Age, department of studies, and students’ marital status were independently related to HPV knowledge score.

However, regarding the possible ways of virus transmission, only 22.1% of students knew all of them, indicating the need for better education. Only 5.9% of students were fully aware of what HPV infections could cause. The percentage of students who knew that HPV causes cervical cancer (56.6%) and genital warts (58.5%) is considered relatively low. Similar results have been observed in previous studies by Kurtinaitiene et al. in which 50.4% of participants knew that HPV causes cervical cancer and 40.6% genital warts [16]. However, in a study conducted on students of Nursing in Turkey, the rate was higher (88%) [17]. In the present survey, only 19.4% of students were aware of all HPV prevention strategies; this percentage was poorer than that reported in another Greek study of Nursing students (48.7%) [12].

However, in a recent study in the USA, the rate of women that had been vaccinated was 46% [18], while in a study conducted in Berlin, Germany, the rate was higher at 67% [19]. In contrast, a lower level towards HPV vaccination was found in a recent study by Monteiro et al. [20], in which 21% of first-year medical students and 16% of 6th-year students had been vaccinated, and also in a Greek study by Vaidakis et al. in which only 10.2% of girls had been vaccinated [21]. According to the results of multivariate linear regression in the present cohort, nationality (*p* = 0.032) and Department of studies (*p* = 0.001) were found to be independently related to the knowledge score about HPV vaccination.

Interestingly, 85% of the sample said that they would like to be vaccinated, a percentage quite satisfactory. This finding was similar to that of the study of Caballero-Perez et al. [22] (87.6%), but also of the study of Jones and Cook (88.6%) [23]. In our study, as the main reason for refusing vaccination, participants reported inadequate information about the vaccine whereas concerns about vaccine side effects emerged as the second most common cause. This finding showed that knowledge and adequate information is a very important predictors; 56.7% of respondents in a similar survey in India answered that inadequate information was the main reason for refusal of vaccination, while 17.6% reported concerns about side effects [24].

Regarding attitudes of female students in screening tests, 97.7% of female students knew what the Pap smear test is. A similar percentage was found in the study of Kamzol et al. [25]. In contrast, a lower result was found in a study by Al-Shaikh et al., where only 46.7% had knowledge of the Pap smear test [26]. Regarding what the Pap smear test detects, 79.7% of female participants knew that the Pap smear test can detect precancerous lesions, a relatively high rate. In contrast, in a corresponding Greek study by Nanou et al., only 51.1% of the sample knew that this test is a diagnostic tool for HPV infection [14]. According to the results of multivariate linear regression, age (*p* = 0.040) and Department of studies (*p* = 0.004) was found to be independent predictors of knowledge about cervical cancer screening.

Finally, according to the results of the study, the percentage of students taking precautions during sexual intercourse was high (85.3%), while 96% of the sample used condoms as a means of protection. The above results converged with a corresponding Greek survey in which the respective percentage was 82.4% [12]. In contrast, in a study by Boyce et al., only 64% of subjects who were sexually active used a condom [27], while in a survey by Denny-Smith et al., the percentage of female students using a condom was lower (37.5%), although 72.9% of them had sexual intercourse [28].

Regarding the limitations, this study was cross-sectional, hence the observed correlations cannot be interpreted as causal. The external validity of the study pertains to the fact that this was a sample of students in two Departments in Crete and cannot be generalized to the entirety of the population of Greek youth. Moreover, this study was performed before the COVID-19 pandemic and therefore further comparative studies would be necessary to evaluate any changes thereafter. Recently, in 2022, the policy of the Greek state changed and offers HPV freely to adolescents of both genders [29]. In addition, this study included only students in Nursing and Social Work; comparative studies versus other healthcare students (e.g., medical doctors) or healthcare professionals would be desirable.

## 5. Conclusions

The findings of the present study identified gaps in student information about HPV, but also highlighted the important role that sociodemographic characteristics and different curricula play in shaping HPV behavior and perception. There is a need for active education and information programs about HPV infection, screening tests, and vaccination. These programs should systematically focus not only on adolescents but also on the entire family, performed by trained educators. The use of health education programs in schools and Universities can be an important measure for the prevention of cervical cancer, minimizing knowledge gaps and improving the existing perceptions of students about HPV and available vaccination. There is a need for comparative studies during the COVID-19 pandemic to evaluate any changes thereafter.

## Figures and Tables

**Table 1:** Sociodemographic characteristics of the study sample.

Variables		*n*	%
**Gender**	Male	61	16.4
	Female	310	83.6
**Age**	18–21	300	80.9
	22–24	36	9.7
	25–29	11	3.0
	>29	24	6.5
**Nationality**	Greek	341	92.4
	Other	28	7.6
**Department of study**	Nursing Department	196	52.8
	Department of Social Work	175	47.2
**Educational level**	First-year students	201	54.2
	Third-year students	170	45.1
**Marital status**	Single	240	65.4
	Married	15	4.1
	Divorced	6	1.6
	In relationship	106	28.9
**Number of siblings**	No	41	11.1
	1	174	47.0
	2	97	26.2
	3	42	11.4
	>3	16	4.3
**Paternal education (years)**	≤6	59	16.6
	7–12	208	58.4
	>12	89	25.0
**Maternal education (years)**	≤6	20	5.6
	7–12	204	56.7
	>12	136	37.8

**Table 2:** Descriptive statistics of knowledge score about HPV, HPV vaccination, and cervical cancer screening.

	Minimum Value	Maximum Value	Mean (SD)	Median (Interquartile Range)
**Knowledge about HPV per se (score)**	0.0	83.3	43.5 (18.7)	41.7 (33.3–58.3)
**Knowledge about HPV vaccination (score)**	0.0	77.3	28.4 (16.2)	27.3 (18.2–40.9)
**Knowledge about cervical cancer screening (score)**	0.0	100.0	76.1 (19.3)	80.0 (60.0–80.0)

**Table 3:** Rate of answers to the questions pertaining to HPV knowledge. Correct answers have been marked in italics.

Questions	Answers	*n*	%	Correct Answer Rate (%)
**Do you know what HPV is?**	No	89	24.1	
	Yes	281	75.9	
**If yes, where have you been informed from?**	Family	110	39.1	
	Friends	64	22.8	
	Television	60	21.4	
	Gynecologist	77	27.4	
	Pediatrician	21	7.5	
	The Press	16	5.7	
	Internet	175	62.3	
	School	150	53.4	
**HPV is a:**	*DNA virus*	75	20.6	20.6
	RNA virus	98	26.9	
	I do not know	191	52.5	
**How can HPV be transmitted?**	Through food	5	1.3	22.1
	Through the air	2	0.5	
	*With sexual contact*	306	82.5	
	*Through skin-to-skin contact between genital organs*	129	34.8	
	*Through contact between genital organs and oral mucosa*	172	46.4	
	This is a hereditary fact	37	10.0	
	I do not know	51	13.7	
**HPV infection can cause**	*Cervical cancer*	210	56.6	5.9
	*Genital warts*	217	58.5	
	*Cancer of the vulva and penis*	98	26.4	
	*Anal cancer*	50	13.5	
	*Oral cancer*	47	12.7	
	I do not know	97	26.1	
**HPV can infect women**	No	7	1.9	83.8
	*Yes*	311	83.8	
	I do not know	53	14.3	
**What are the two HPV serotypes that have been associated with a significant increase in cervical cancer risk?**	*16 and 18*	31	8.4	8.4
	11 and 6	5	1.4	
	42 and 44	12	3.3	
	31 and 33	14	3.8	
	I do not know	306	83.2	
**An infected person can be asymptomatic**	No	44	11.9	56.3
	*Yes*	209	56.3	
	I do not know	118	31.8	
**HPV can spontaneously resolve without treatment**	No	285	76.8	3.0
	*Yes*	11	3.0	
	I do not know	75	202	
**Sexual contact with multiple partners can increase the risk of HPV infection**	No	16	4.3	83.0
	*Yes*	307	83.0	
	I do not know	47	12.7	
**How can HPV infection be prevented?**	*Through avoidance of sexual contact with multiple sex partners*	96	25.9	19.4
	*Through condom use*	274	73.9	
	*Through HPV vaccination*	220	59.3	
	I do not know	44	11.9	
**HPV can infect men**	No	24	6.5	74.7
	*Yes*	277	74.7	
	I do not know	70	18.9	
**HPV can be detected by molecular biology tests**	No	2	0.5	67.6
	*Yes*	250	67.6	
	I do not know	118	31.9	

**Table 4:** Multivariate linear regression analysis of log-transformed knowledge scores about HPV per se, in association with sociodemographic features. Bold cells denote statistically significant associations.

Variables	Categories	Beta *	SE ^§^	*p*
**Sex**	Male (reference)			
	Female	0.02	0.06	0.723
**Age (years)**	18–21 (reference)			
	>21	0.28	0.09	**0.002**
**Nationality**	Greek (reference)			
	Other	0.11	0.06	0.067
**Department of studies**	Department of Nursing (reference)			
	Department of Social Work	−0.16	0.04	**<0.001**
**Marital status**	Married (reference)			
	Not married	−0.10	0.04	**0.015**
**Number of siblings**	Increments of one	−0.01	0.03	0.624
**Paternal education (years)**	Increments of one	0.09	0.05	0.065
**Maternal education (years)**	Increments of one	−0.04	0.04	0.382

* Regression coefficient; § standard error.

**Table 5:** HPV vaccination status, choice of HPV vaccination, and reasons for taking the vaccine.

Variables	Categories	*n*	%
**HPV vaccination**	No	242	66.3
	Yes	123	33.7
**If yes, who prompted you to receive the vaccine?**	Family	84	68.3
	Friend	6	4.9
	Gynecologist	28	22.8
	Pediatrician	64	52.0
	The Press	1	0.8
	Television	2	1.6
	Internet	8	6.5
	School	4	3.3
**Which vaccine have you been vaccinated with?**	Bivalent	6	4.9
	Quadrivalent	27	22.1
	Nine-valent	8	6.6
	Do not know	81	66.4

**Table 6:** Multivariate linear regression analysis of log-transformed knowledge scores about HPV vaccination, in association with sociodemographic features. Bold cells denote statistically significant associations.

Variables	Categories	Beta *	SE ^§^	*p*
**Gender**	Male (reference)			
	Female	0.08	0.06	0.136
**Age (years)**	18-21 (reference)			
	>21	0.07	0.06	0.208
**Nationality**	Greek (reference)			
	Other	−0.19	0.09	**0.032**
**Department of studies**	Department of Nursing (reference)			
	Department of Social Work	−0.15	0.04	**0.001**
**Marital status**	Married (reference)			
	Not married	−0.04	0.05	0.373
**Number of siblings**	Increments of one	−0.03	0.03	0.300
**Paternal education (years)**	Increments of one	0.06	0.04	0.126
**Maternal education (years)**	Increments of one	−0.03	0.04	0.500

* Regression coefficient; § standard error.

**Table 7:** Multivariate linear regression analysis of log-transformed knowledge scores about cervical cancer screening, in association with sociodemographic features; this analysis was confined to female students. Bold cells denote statistically significant associations.

Variables	Categories	Beta *	SE ^§^	*p*
**Age (years)**	18-21 (reference)			
	>21	0.09	0.04	**0.040**
**Nationality**	Greek (reference)			
	Other	0.04	0.06	0.537
**Department of studies**	Department of Nursing (reference)			
	Department of Social Work	−0.09	0.03	**0.004**
**Marital status**	Married (reference)			
	Not married	−0.04	0.03	0.170
**Number of siblings**	Increments of one	−0.03	0.02	0.088
**Paternal education (years)**	Increments of one	0.01	0.03	0.593
**Maternal education (years)**	Increments of one	0.01	0.03	0.644

* Regression coefficient; § standard error.

**Table 8:** Multivariate logistic regression analysis of having a Pap smear test in association with sociodemographic features; this analysis was confined to female students. Bold cells denote statistically significant associations.

		OR (95% CI)	*p*
**Age (years)**	18-21 (reference)		
	>21	0.58 (0.15–2.25)	0.429
**Nationality**	Greek (reference)		
	Other	Not estimable due to small numbers	
**Department of studies**	Department of Nursing (reference)		
	Department of Social Work	0.18 (0.04–0.72)	**0.015**
**Marital status**	Married (reference)		
	Not married	0.44 (0.12–1.65)	0.224
**Number of siblings**	Increments of one	0.68 (0.34–1.37)	0.286
**Paternal education (years)**	Increments of one	0.63 (0.22–1.82)	0.389
**Maternal education (years)**	Increments of one	1.26 (0.44–3.61)	0.667

## Data Availability

Not applicable.
